# Dermal fibroblast expression of stromal cell-derived factor-1 (SDF-1) promotes epidermal keratinocyte proliferation in normal and diseased skin

**DOI:** 10.1007/s13238-015-0198-5

**Published:** 2015-08-22

**Authors:** Chunji Quan, Moon Kyun Cho, Yuan Shao, Laurel E. Mianecki, Eric Liao, Daniel Perry, Taihao Quan

**Affiliations:** Department of Pathology, Affiliated Hospital of Yanbian University, Yanji, 133000 China; Department of Dermatology, Soonchunhyang University College of Medicine, Seoul, Republic of Korea; Hollings Cancer Center Biorepository and Tissue Analysis Resource, Medical University of South Carolina, Charleston, SC 29425 USA; Department of Dermatology, University of Michigan Medical School, Ann Arbor, MI 48109 USA

**Keywords:** SDF-1, dermal fibroblast, keratinocyte, proliferation, skin cancer, psoriasis

## Abstract

Stromal cells provide a crucial microenvironment for overlying epithelium. Here we investigated the expression and function of a stromal cell-specific protein, stromal cell-derived factor-1 (SDF-1), in normal human skin and in the tissues of diseased skin. Immunohistology and laser capture microdissection (LCM)-coupled quantitative real-time RT-PCR revealed that SDF-1 is constitutively and predominantly expressed in dermal stromal cells in normal human skin *in vivo*. To our surprise, an extremely high level of SDF-1 transcription was observed in the dermis of normal human skin *in vivo*, evidenced by much higher mRNA expression level than type I collagen, the most abundant and highly expressed protein in human skin. SDF-1 was also upregulated in the tissues of many human skin disorders including psoriasis, basal cell carcinoma (BCC), and squamous cell carcinoma (SCC). Double immunostaining for SDF-1 and HSP47 (heat shock protein 47), a marker of fibroblasts, revealed that fibroblasts were the major source of stroma-cell-derived SDF-1 in both normal and diseased skin. Functionally, SDF-1 activates the ERK (extracellular-signal-regulated kinases) pathway and functions as a mitogen to stimulate epidermal keratinocyte proliferation. Both overexpression of SDF-1 in dermal fibroblasts and treatment with rhSDF-1 to the skin equivalent cultures significantly increased the number of keratinocyte layers and epidermal thickness. Conversely, the stimulative function of SDF-1 on keratinocyte proliferation was nearly completely eliminated by interfering with CXCR4, a specific receptor of SDF-1, or by knock-down of SDF-1 in fibroblasts. Our data reveal that extremely high levels of SDF-1 provide a crucial microenvironment for epidermal keratinocyte proliferation in both physiologic and pathologic skin conditions.

**Electronic supplementary material:**

The online version of this article (doi:10.1007/s13238-015-0198-5) contains supplementary material, which is available to authorized users.

## Introduction

Human skin is the largest organ of the human body, and is composed mainly of two layers: the epidermis (outermost layer) and the dermis (vascular connective tissue below the epidermis). The epidermis is primarily composed of keratinocytes and the dermis is composed of a dense collagen-rich extracellular matrix (ECM) and stromal cells such as fibroblasts. The complex organization of skin is designed to support the skin’s numerous functions, including forming a protective barrier from environmental stresses such as water loss and microorganism infection.

In human skin the dermis not only provides the structural and mechanical properties of the skin (Uitto, 1986), but also intimately interacts with the epidermis to maintain skin homeostasis (Maas-Szabowski et al., 1999; Tuan et al., 1994). Interactions between mesenchymal stromal cells and epithelial cells play an important role in regulating tissue development, homeostasis, regeneration, and tumorigenesis (Donjacour and Cunha, 1991). In human skin, stromal cells such as dermal fibroblasts provide a crucial microenvironment for epidermal keratinocyte function (Blanpain and Fuchs, 2009). However, not much is known about the molecular details and the factors responsible for such complex interactions between dermal stromal cells and epidermal keratinocytes in both physiologic and pathologic conditions.

In this study we aimed to examine the expression and function of stromal cell-derived factor-1 (SDF-1 or CXCL12), a chemokine that is secreted by stromal cells and acts on neighboring cells. SDF-1 has been found to play an important role in inflammation, proliferation, tumorigenicity, and metastasis (Kryczek et al., 2007; Luker and Luker, 2006; Orimo et al., 2005). For example, SDF-1 promotes tumor growth through stimulation of angiogenesis and by secretion of several mitogens (Chen et al., 2006; Chu et al., 2009; Wang et al., 2005). However, the role and expression of SDF-1 in normal human skin and skin disorders have not been comprehensively documented. Here we demonstrate that SDF-1 is constitutively and predominantly expressed in normal human skin dermis with extremely high expression levels. Moreover, SDF-1 is greatly upregulated in pathological conditions of human skin including psoriasis and keratinocyte skin cancers, BCC and SCC. Functional studies indicate that SDF-1 is mainly secreted by dermal fibroblasts and functions as a mitogen for epidermal keratinocyte proliferation through activation of the ERK/MAPK pathway, the predominant growth pathway in skin keratinocytes (Chambard et al., 2007; Eckert et al., 2002; Jost et al., 2001).

## Results

### SDF-1 is substantially expressed in normal human skin

**Figure 1:**
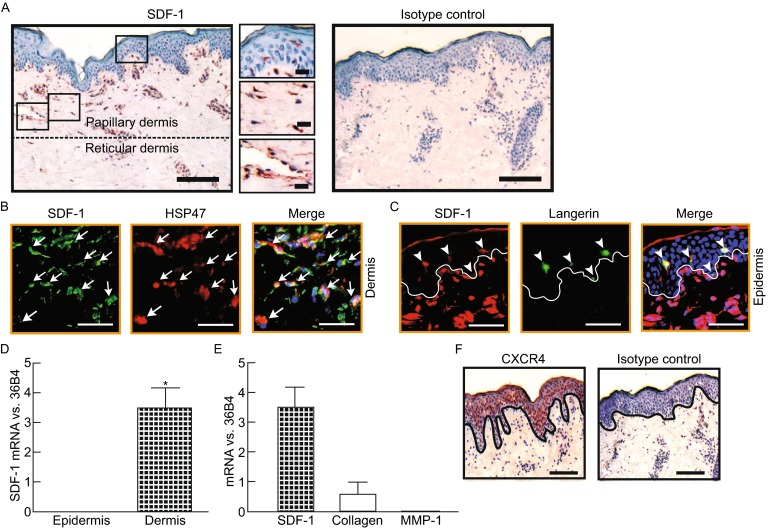
**SDF-1 is predominantly expressed in normal human skin dermis with extremely high levels**. (A) SDF-1 protein expression and localization in normal human skin. OCT-embedded skin sections (7 μm) were immunostained with SDF-1 antibody (left) and isotype control antibody (mouse IgG1, right). Bar = 100 µm. 2.5× enlargement of the boxed region is shown to the right (top, epidermis; middle, fibroblasts; lower, vascular endothelial cells, bar = 50 µm). Representative of six individuals. (B) The majority of SDF-1 positive cells were dermal fibroblasts. OCT-embedded skin sections (7 μm) were co-immunofluoresce stained with SDF-1 and HSP47, a marker of dermal fibroblast. Arrows indicate double stained cells. Representative of five individuals. Bar = 50 µm. (C) Langerhans cells were stained with SDF-1 in epidermis. OCT-embedded skin sections (7 μm) were co-immunofluoresce stained with SDF-1 and Langerin, a marker of langerhans cell. Arrow heads indicate double stained cells. Lines indicate epidermal and dermal junctions. Representative of six individuals. Bar = 50 µm. (D) An extremely high level of SDF-1 mRNA expression in dermis was confirmed by Laser capture microdisection (LCM) coupled quantitative real-time RT-PCR. Epidermis and dermis were captured by LCM. Total RNA was extracted from captured tissue, and mRNA levels were quantified by real-time RT-PCR. SDF-1 mRNA levels were normalized to the housekeeping gene 36B4 as an internal control for quantification. Data are relative levels to 36B4 (mean ± SEM), *n* = 6, *P = 0.001. (E) SDF-1 mRNA expression is higher than type I collagen mRNA expression. Total RNA was extracted from LCM-captured dermis and mRNA levels were quantified by real-time RT-PCR. The target gene expression levels were presented relative to an internal control (36B4). Data are relative levels to 36B4 (mean ± SEM), *n* = 6–10. (F) CXCR4 protein expression and localization in normal human skin. OCT-embedded skin sections (7 μm) were immunostained with CXCR4 antibody (left) and isotype control antibody (mouse IgG1, right). Lines indicate epidermal and dermal junction. Representative of six individuals. Bar = 100 µm

Although SDF-1 expression in human skin has been reported (Pablos et al., 1999), its expression levels, patterns, and cell type-specific expression in normal human skin compartments remain incompletely understood. Immunostaining for SDF-1 on sections of normal human skin revealed that SDF-1 expression was largely restricted to stromal cells in the dermal compartment (Fig. 1A), such as dermal fibroblasts (Fig. 1A, middle insert) and vascular endothelial cells (Fig. 1A, lower insert). Interestingly we noticed that SDF-1 staining was much higher in papillary dermal cells, which reside in the superficial dermis, than in reticular dermal cells. No staining was detected with isotype matched control IgG (Fig. 1A, right). Double immunostaining for SDF-1 and HSP47, a marker of fibroblasts, revealed co-localization indicating that the majority of SDF-1 positive cells were dermal fibroblasts (Fig. 1B). In addition to dermal stromal cells, scattered epidermal cells were also stained with SDF-1 (Fig. 1A, top insert). Double immunostaining for SDF-1 and langerin, a marker of langerhans cells, revealed co-localization indicating that the scattered SDF-1 positive cells in the epidermis were langerhans cells (Fig. 1C). Next we determined the relative mRNA expression levels of SDF-1 in the epidermis and dermis by laser capture microdissection-coupled real-time RT-PCR (Qin et al., 2013) (Fig. 1D). Surprisingly we found that SDF-1 mRNA levels were extremely high in the dermis of normal human skin, evidenced by much higher mRNA level than the housekeeping gene mRNA, 36B4 (Fig. 1D). Further investigation indicated that the SDF-1 mRNA level was much higher than mRNA level of type I collagen (Fig. 1E), the most abundant protein in human skin, and matrix metallopriteianse-1 (MMP-1), which has been shown to have a very low expression level in normal skin (Quan et al., 2009). Next we investigated the localization of CXCR4, the specific receptor for SDF-1, in human skin. As shown in Fig. 1F, CXCR4 was predominantly expressed in human skin epidermis. Taken together, these data demonstrate that SDF-1 is constitutively and extremely highly expressed in normal human skin dermis, suggesting SDF-1 may play an important role in skin homeostasis in physiological conditions.

### SDF-1 is overexpressed in human keratinocyte skin cancers

**Figure 2:**
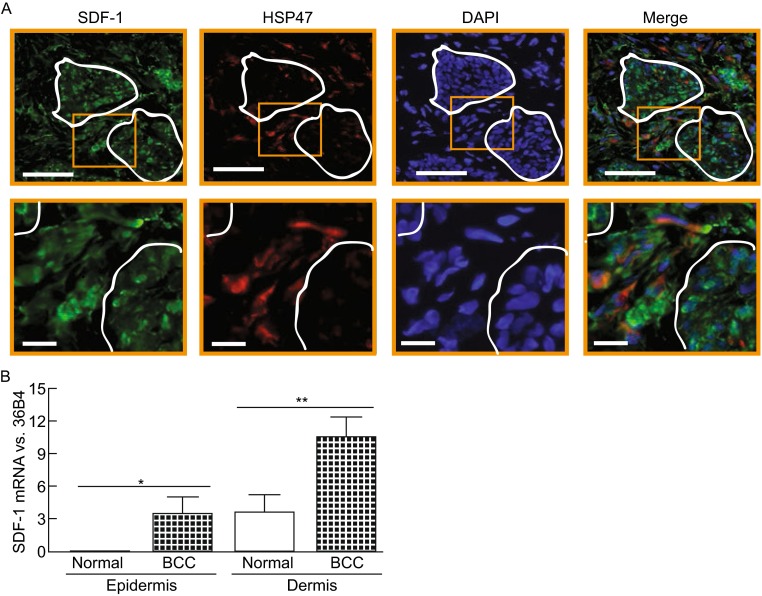
**SDF-1 is overexpressed in human skin basal cell carcinoma (BCC)**. (A) SDF-1 immunostaining in human BCC. OCT-embedded BCC sections (7 μm) were co-immunofluoresce stained with SDF-1 and HSP47. Circles indicate BCC islands. Representative of six BCC tissues. Bar = 100 µm. 2.5× enlargement of the boxed region is shown to the bottom (bar = 50 µm). Representative of six individuals. (B) SDF-1 mRNA expression in BCC and BCC-associated stromal tissue. BCC, BCC-associated stromal tissue, normal epidermis and dermis were captured by LCM. Total RNA was extracted from captured tissues, and mRNA levels were quantified by real-time RT-PCR. SDF-1 mRNA levels were normalized to the housekeeping gene 36B4 as an internal control for quantification. Data are relative levels to 36B4 (mean ± SEM), *n* = 12, *P = 0.007, **P = 0.02

**Figure 3:**
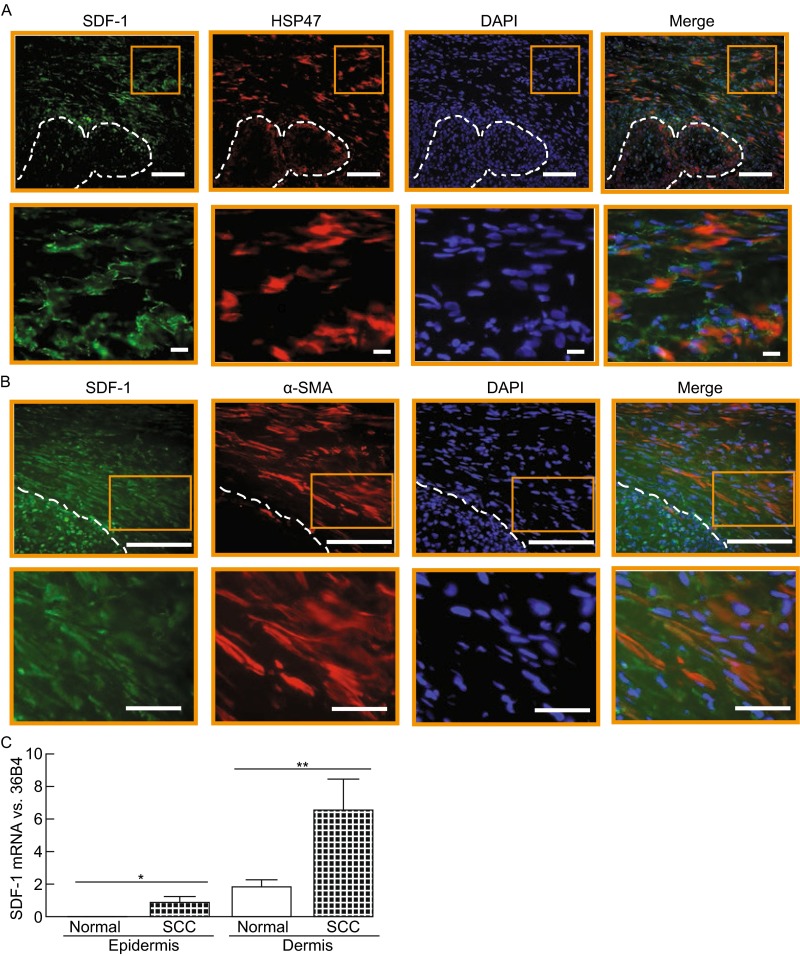
**SDF-1 is overexpressed in human skin squamous cell carcinoma (SCC)**. (A) SDF-1 immunostaining in human SCC. OCT-embedded SCC sections (7 μm) were co-immunofluoresce stained with SDF-1 and HSP47. Bar = 100 µm. Outlines indicate SCC islands. 3.5× enlargement of the boxed region is shown to the bottom (bar = 50 µm). Representative of six SCC tissues. (B) Double immunostaining for SDF-1 and α-smooth muscle actin (α-SMA). Outlines indicate SCC islands. Representative of six SCC tissues. Bar = 100 µm. 2× enlargement of the boxed region is shown to the bottom (bar = 50 µm). Representative of six individuals. (C) SDF-1 mRNA expression in SCC and SCC-associated stromal tissue. SCC, SCC-associated stromal tissue, normal epidermis and dermis were captured by LCM. Total RNA was extracted from captured tissues, and mRNA levels were quantified by real-time RT-PCR. SDF-1 mRNA levels were normalized to the housekeeping gene 36B4 as an internal control for quantification. Data are relative levels to 36B4 (mean ± SEM), *n* = 12, *P = 0.03, **P = 0.04

We next examined SDF-1 expression in human skin BCC and SCC, two major keratinocyte skin cancers. SDF-1 immunostaining of BCC skin sections revealed a much greater cellular staining in BCC-associated stroma (Fig. 2A). Interestingly, BCC keratinocytes were also positively stained with SDF-1, while in normal human skin keratinocytes were completely negative (Fig. 1A). Double immunostaining for SDF-1 and HSP47 revealed co-localization, indicating the majority of SDF-1 positive cells were fibroblasts in the BCC stroma. LCM-coupled quantitative real-time RT-PCR indicated that the SDF-1 mRNA levels were significantly elevated in BCC and BCC-associated stroma compared to normal epidermis and dermis, respectively (Fig. 2B). SDF-1 immunostaining of SCC skin sections resulted in similar patterns, showing significantly elevated SDF-1 levels in SCC-associated stroma and SCC (Fig. 3A). Double immunostaining for SDF-1 and HSP 47 confirmed the co-localization, indicating that fibroblasts were expressing SDF-1 in the SCC stroma. Additionally, the majority of SDF-1 positive cells in SCC-stroma were co-localized with α-smooth muscle actin (α-SMA), a marker for myofibroblasts (Fig. 3B). LCM-derived SCC and stromal tissue indicated that SDF-1 mRNA levels were significantly elevated in both SCC and SCC-associated stromal tissues compared to normal epidermis and dermis, respectively (Fig. 3C). These data demonstrate that SDF-1 is significantly upregulated in both BCC/SCC-associated stromal fibroblasts and BCC/SCC keratinocytes.

### SDF-1 expression is up-regulated in human psoriatic skin

**Figure 4:**
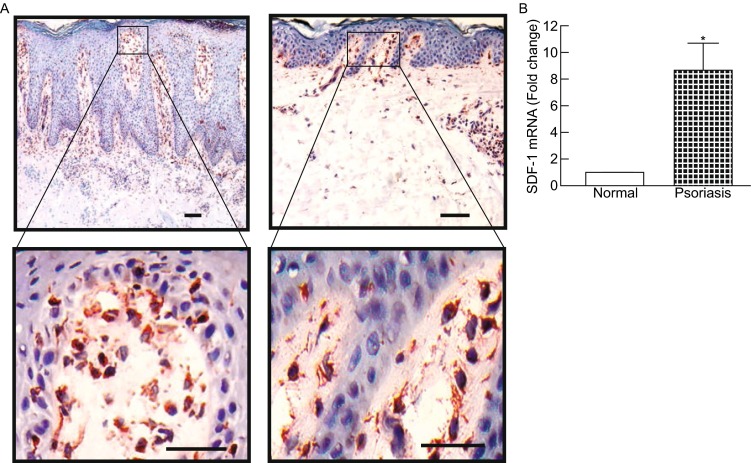
**SDF-1 is overexpressed in human psoriatic skin**. (A) SDF-1 immunostaining in human psoriatic skin. OCT-embedded psoriatic sections (7 μm) were stained with SDF-1. Bar = 100 µm. 7.5× (left) and 4.5× (right) enlargement of the boxed regions are shown to the bottom (bar = 50 µm). Representative of six individuals. (B) SDF-1 mRNA expression in psoriatic skin. Total RNA was extracted from psoriatic tissue or non-lesional normal skin in the same patients. SDF-1 mRNA levels were quantified by real-time RT-PCR and normalized to the housekeeping gene 36B4 as an internal control for quantification. Data are relative levels to 36B4 (mean ± SEM), *n* = 6, *P = 0.02

Next we investigated SDF-1 expression in skin of patients with psoriasis, a common chronic inflammatory skin disorder. Immunostaining of sections of human psoriatic tissue indicated increased SDF-1 staining which was largely restricted to the dermal cells and scattered epidermal cells, presumably langerhans cells (Fig. 4A). Interestingly, SDF-1 was most highly expressed at the dermal-epidermal junction in both psoriatic lesional tissue (Fig. 4A, left) and adjacent tissue (Fig. 4A, right). Quantification indicated that the SDF-1 mRNA level was increased 8.2-fold in psoriatic tissue compared to non-lesional normal skin in the same patients.

### SDF-1 activates ERK pathway and enhances keratinocyte proliferation

**Figure 5:**
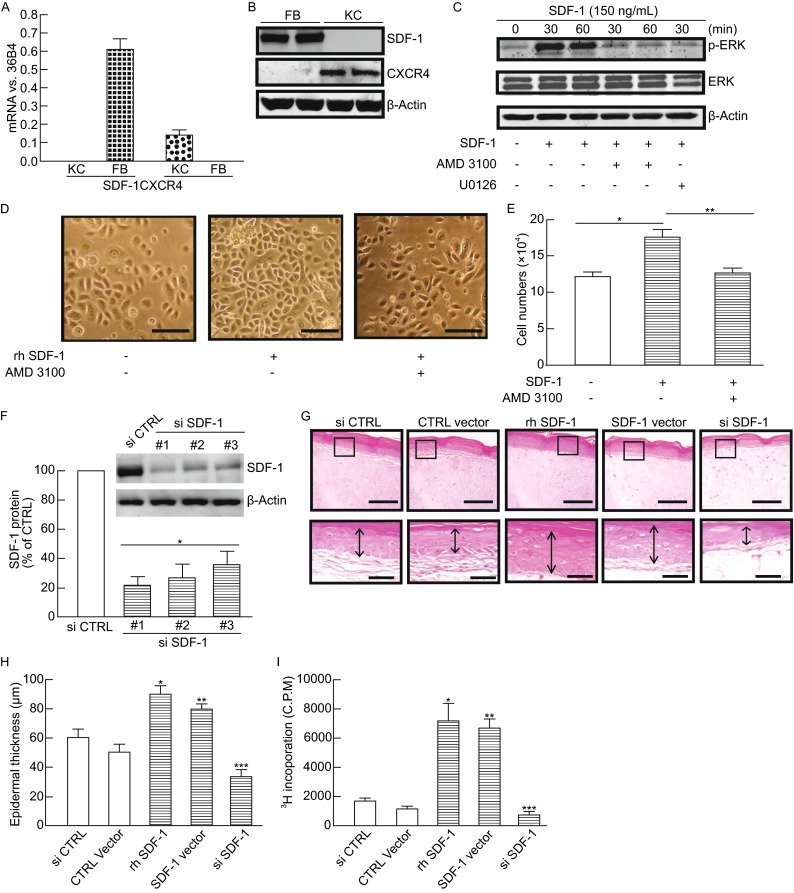
**SDF-1 activates ERK pathway and functions as a mitogen to promote keratinocyte proliferation**. (A) SDF-1 mRNA and its receptor CXCR4 were specifically expressed in fibroblasts and keratinocytes, respectively. Total RNA was extracted from primary dermal fibroblasts and keratinocytes. SDF-1 and CXCR4 mRNA levels were quantified by real-time RT-PCR and normalized to the housekeeping gene 36B4 as an internal control for quantification. Data are relative levels to 36B4 (mean ± SEM), *n* = 4. (B) SDF-1 protein and its receptor CXCR4 were specifically expressed in fibroblasts and keratinocytes, respectively. Whole cell extract was prepared from primary dermal fibroblasts and keratinocytes. Protein levels were determined by Western analysis. *n* = 2. (C) SDF-1 rapidly activated the ERK1/2 in primary keratinocytes, and that was reduced by CXCR4 (ADM3100) and MEK (U0126) inhibitors. Cells were pretreated with CXCR4 or MEK inhibitors or remained untreated (control). These cells were then treated with rh SDF-1 (150 ng/mL) at indicated times. Whole cell extract was prepared and protein levels were determined by Western analysis. *n* = 3. (D) SDF-1 promotes keratinocytes proliferation. Cells were pretreated with CXCR4 antagonist (ADM3100) or PBS (control). These cells were then treated with rh SDF-1 (150 ng/mL). Five days after treatment, cells were photographed (phase-contract microscopy), harvested, and counted using a hemocytometer. Bar = 100 µm. (E) Results are means ± SEM, *n* = 3. *P = 0.04, **P = 0.03. Bar = 100 µm. (F) Efficiency of knockdown by SDF-1 siRNAs. SDF-1 protein levels were determined 2 days after transfection with SDF-1 siRNAs by Western blot analysis. Band intensities were quantified by chemifluorescence and normalized to internal control β-actin. The insert shows a representative Western blot. Data are means ± SEM. *n* = 3. *P = 0.05, (G) Skin equivalent cultures were prepared as described in “Materials and methods”. Dermal fibroblasts were infected with control adenovirus (second left) or premade adenovirus SDF-1 (second right) before being embedded in collagen lattices. Skin equivalent culture was treated with rh SDF-1 (200 ng/mL) for seven days (middle). Air-lifted skin equivalent cultures were harvested and embedded in OCT, and cryo-sections were stained with H&E. Bar = 100 µm. 4.6× enlargement of the boxed regions are shown to the bottom (bar = 25 µm). Representative of three experiments. (H) The epidermal thickness was measured by computerized image analysis. Results are means ± SEM, *n* = 4. *P = 0.03 vs. CTRL, **P = 0.01 vs. CTRL vector, ***P = 0.01 vs. si CTRL. (I) Proliferation of epidermal keratinocytes was determined by [^3^H]thymidine incorporation assay, as described in “Materials and methods”. Results are means ± SEM, *n* = 3. *P = 0.03 vs. CTRL, **P = 0.02 vs. CTRL vector, ***P = 0.05 vs. si CTRL

Prior human skin *in vivo* studies indicated that SDF-1 is predominantly expressed in the dermis of normal human skin and significantly elevated in the pathological conditions of several skin disorders. These data prompted us to investigate the role of SDF-1 in human skin. To this end, we first investigated the expression of SDF-1 and its receptor CXCR4 in two major cell types in human skin, dermal fibroblasts and epidermal keratinocytes. In agreement with our human skin *in vivo* results (Fig. 1A), we confirmed that SDF-1 mRNA was specifically expressed in primary human dermal fibroblasts but not in primary human keratinocytes (Fig. 5A). In contrast to SDF-1, keratinocytes, but not the fibroblasts, expressed the receptor CXCR4. Western analysis further confirmed that SDF-1 and its receptor CXCR4 are specifically expressed in fibroblasts and keratinocytes, respectively (Fig. 5B). Since SDF-1/CXCR4 interaction has been shown to activate several signaling pathways including MAPK in various cell lines (Chu et al., 2009; Kijima et al., 2002), we performed Western blot analysis to elucidate ERK activation, a predominant growth pathway in skin keratinocytes (Chambard et al., 2007; Eckert et al., 2002; Jost et al., 2001). SDF-1 rapidly activated the ERK1/2 pathway in primary keratinocytes as evidenced by elevated phosphorylation of p42 and p44 (p-ERK1/2) (Fig. 5C), which was greatly reduced by ADM3100, a highly specific CXCR4-antagonist, and U0126, a specific MEK inhibitor. In agreement with ERK activation, addition of recombinant human SDF-1 to the keratinocyte culture medium resulted in increased keratinocyte proliferation (Fig. 5D) and cell number quantification (Fig. 5E). The role of SDF-1 as a mitogen for primary keratinocytes was further demonstrated by interfering with a specific CXCR4-antagonist, ADM3100 (Fig. 5D and 5E). Next we investigated the role of SDF-1 in keratinocyte proliferation using gain- and loss-of-function approaches. Figure 5F shows the knockdown efficiency of SDF-1 (see Figs. S1 and S2 for detailed information of off-target effects). Among three SDF-1 siRNAs tested, #1 SDF-1 siRNA showed the highest knockdown efficiency, therefore, we utilized #1 SDF-1 siRNA in following experiments. To test epidermal keratinocyte proliferation, we employed a skin equivalent culture system, which is a well-characterized model to study the interaction of epidermal keratinocytes and dermal fibroblasts (Boelsma et al., 1999; Pickard et al., 2012; Ridky et al., 2010). Skin equivalent cultures are composed of stratified, differentiated keratinocytes (model epidermis) on top of a type I collagen lattice in which dermal fibroblasts are embedded (model dermis). Both overexpression of SDF-1 in dermal fibroblasts and treatment with rhSDF-1 to the skin equivalent cultures significantly increased the number of keratinocyte layers (Fig. 5G). In contrast, knock-down SDF-1 levels in dermal fibroblasts resulted in a significant inhibition of the number of keratinocyte layers. Quantification indicated that the thickness of the keratinocyte layers was increased by more than 150% (Fig. 5H) with either overexpression of SDF-1 in dermal fibroblasts or treatment with rhSDF-1. In contrast, knock-down SDF-1 levels in dermal fibroblasts reduced the thickness of the keratinocyte layers by 33%. To further assess the proliferation of epidermal keratinocytes, we performed a thymidine incorporation assay by directly measuring DNA synthesis in mitotic keratinocytes. In agreement with epidermal thickness (Fig. 5G and 5H), DNA synthesis was significantly increased by either rhSDF-1 or overexpression of SDF-1 in dermal fibroblasts, compared to control. In contrast, knock-down SDF-1 levels in dermal fibroblasts reduced DNA synthesis. Taken together, these data demonstrate that SDF-1 is secreted from dermal fibroblasts and functions as a mitogen for epidermal keratinocytes.

## Discussion

Surprisingly, we found that basal expression of SDF-1 mRNA levels were extremely high in normal human skin dermis *in vivo*. SDF-1 mRNA levels were several folds higher than type I collagen mRNA, the most abundant ECM protein in human skin. Given that the SDF-1 mRNA level is even greater than collagen, SDF-1 may play an important role in skin homeostasis under physiological conditions. This notion is supported by our data that fibroblast-derived SDF-1 functions as a mitogen to stimulate keratinocyte proliferation and thus may contribute to epidermal turnover and morphogenesis. In normal skin the epidermis constantly turns over at a high rate, averaging 28 days in humans and 7 days in mice (Potten, 1981). This constantly high level of epidermal turnover is mediated by epidermal stem cells (Koster, 2009), which reside in the basal layer of the epidermis. Stem cells in the epidermis have a crucial role in maintaining tissue homeostasis by providing new cells to replace those that are constantly lost during tissue turnover. Given that SDF-1 functions as a mitogen to stimulate epidermal keratinocyte proliferation, it is conceivable that SDF-1 might be involved in epidermal stem cell survival and proliferation that contribute to epidermal turnover. In agreement with this notion, SDF-1 has been shown to play an important role in hematopoietic and progenitor stem cell motility and development in both human and murine (Koster, 2009; Wright et al., 2002). SDF-1 is highly expressed in the bone marrow by various stromal cells including osteoblasts, endothelial cells, and perivascular reticular cells (Lapidot et al., 2005; Sugiyama et al., 2006). Notably, SDF-1 is also involved in other stem cell functions including regulation of cell quiescence and survival (Nie et al., 2008; Sugiyama et al., 2006). Our data suggest that extremely high levels of SDF-1 in the dermis might be required for skin homeostasis, including epidermal turnover and morphogenesis. Notably normal human skin does not display overt inflammation, suggesting that SDF-1 may function as an important mitogen in physiological conditions rather than as a chemokine, a well-known major function of SDF-1 which activates leukocytes and is often induced by proinflammatory stimuli. Nevertheless, future studies must be carried out to determine how SDF-1 does play a role in skin homeostasis, epidermal turnover, and morphogenesis under physiological conditions.

Interestingly, Takekoshi et al. recently reported that SDF-1/CXCR4 signaling normally limits keratinocyte proliferation instead of stimulation in keratinocyte-specific CXCR4 knockout mouse models (Nishimura et al., 2012). Some existing literatures support this concept, whereas others contradict it (Bollag and Hill, 2013). Obviously, the precise roles of the SDF-1/CXCR4 axis in human skin appear to be complex. It is worth mentioning that a second SDF-1 receptor, CXCR7, has been recently identified (Duda et al., 2011). Indeed, some of the functions of SDF-1 are mediated through CXCR7 rather than CXCR4. CXCR7 has also been implicated in regulating cell proliferation, adhesion, and migration (reviewed in Maksym et al., 2009). Additionally, AMD3100, a known CXCR4 inhibitor, appears to act as an agonist at the CXCR7 receptor (Kalatskaya et al., 2009), suggesting that additional investigation of this important SDF-1/CXCR4/CXCE7 axis in skin biology is clearly warranted.

The critical role of dermal fibroblasts in regulating epidermal morphogenesis has been reported (El Ghalbzouri et al., 2002). For example, in the absence of fibroblasts the epidermis formed very thin cell and stratum corneum layers. However, in the presence of fibroblasts the proliferation and migration of keratinocytes was stimulated and epidermal morphology was markedly improved. These data suggest that fibroblasts are required for epidermal morphogenesis by providing growth factors needed for the generation of a fully differentiated epidermis. This notion is supported by our recent evidence demonstrating that improvement of aged dermal fibroblast function *in vivo* significantly increased epidermal keratinocyte proliferation and thus increased epidermal thickness, which is a prominent feature of aged human skin (Quan et al., 2013). Comparing SDF-1 expression in papillary and reticular dermal fibroblasts, we noticed that papillary dermal fibroblasts expressed much higher SDF-1 levels than reticular dermal fibroblasts, suggesting papillary dermal fibroblasts are the major source of SDF-1 and are actively involved in epidermal morphogenesis. In agreement with this notion, Mine et al. reported that dermal equivalents containing papillary dermal fibroblasts were more potent in promoting epidermal morphogenesis than those containing reticular dermal fibroblasts (Mine et al., 2008).

Although SDF-1 expression is extremely high in normal human skin, its expression is even greater in human skin keratinocyte hyperproliferative disorders. We found that SDF-1 positive stromal cells are restricted to the dermal-epidermal junction in both psoriatic lesional tissue and adjacent tissue, suggesting stromal cell-derived SDF-1 may play an important role in the development of chronic skin inflammation. The hallmark of psoriasis is hyperproliferation of keratinocytes leading to thickening of the epidermis and elongated rete ridges that form fingerlike protrusions into the dermis. Given the stimulative function of SDF-1 on epidermal keratinocyte proliferation, it is imaginable that elevated stromal cell-derived SDF-1 might be actively involved in the hallmark of psoriasis, hyperproliferation of keratinocytes and thickening of the epidermis. This concept has been investigated in experimental models of chronic psoriasis-like skin inflammation, which reveal an important role of the SDF-1/CXCR4 axis in skin inflammation (Zgraggen et al., 2014).

Elevated expression of SDF-1 has been reported in a number of cancer-associated stroma (Kryczek et al., 2007), including keratinocyte skin cancers (Chen et al., 2009). This study demonstrates the expression of SDF-1 in tumor stromal fibroblasts and appendages, but not in BCC epithelial cancer cells. In contradiction, we found that in keratinocyte skin cancers epithelial cancer cells are positively stained with SDF-1, while in normal human skin keratinocytes are completely negative. It is possible that the high level of immunostaining of SDF-1 in cancer cells resulted from accumulation of secreted SDF-1 by stromal cells through SDF-1/CXCR4 interaction. However, LCM-captured BCC/SCC islands further confirmed the high expression of SDF-1 transcription in epithelial cancer cells compared to normal skin epidermis. The reasons for this discrepancy are not clear at this time. Although the nature of SDF-1 positive epithelial cells in keratinocyte skin cancers is not known, one possibility is that SDF-1 may promote epithelial-mesenchymal transition (EMT) and thus they express stromal marker SDF-1. In agreement with this notion, we observed HSP47, a marker of stromal fibroblasts, positive cells surrounding BCC (Fig. 2) and SCC (Fig. 3) islands, indications of EMT. EMT refers to critical events in tumor progression including invasion and metastasis by which cancer cells acquire a fibroblast-like phenotype. Indeed, it has been reported that SDF-1/CXCR4 promotes EMT and progression of colorectal cancer (Hu et al., 2014), oral squamous cell carcinoma, (Onoue et al., 2006), hepatocellular carcinoma (Li et al., 2014), and pancreatic cancer cells (Li et al., 2012). Nevertheless, cancer-associated stromal cells are heavily stained with SDF-1 suggesting an important role of stromal cell-derived SDF-1 in skin cancer progression including the possibility of promoting cancer cell EMT.

It is becoming increasingly clear that stromal microenvironments play a critical role in tumor development and progression (Mueller and Fusenig, 2004; Quail and Joyce, 2013). Particularly, research shows that activated fibroblasts, often referred to as carcinoma-associated-fibroblasts (CAFs), play an important role in promoting tumorigenesis (Bhowmick et al., 2004; Straussman et al., 2012). Orimo et al. reports that CAFs from human breast carcinomas aid the growth of tumor cells by secreting SDF-1. Their study shows that CAFs produce SDF-1 which in turn mediates tumor angiogenesis by recruiting endothelial progenitor cells (Orimo et al., 2005). In agreement with this, we found significantly increased angiogenesis in both BCC and SCC (Fig. S1), suggesting that stromal cell derived SDF-1 not only functions as a mitogen to stimulate epithelial cancer cell growth, but also to promote tumor angiogenesis.

We found that the majority of SDF-1 positive cells are dermal fibroblasts in both normal and diseased skin. Dermal fibroblasts are the most populated stromal cells in human skin. It is well-known that the major function of dermal fibroblasts is to synthesize collagen-rich ECM (Farage et al., 2010; Fisher et al., 2008). Dermal fibroblasts secrete the precursors of all the components of the ECM proteins in skin. Therefore, the main function of dermal fibroblasts is to maintain the structural and mechanical integrity of dermal connective tissue by continuously synthesizing and secreting collagen and other ECM proteins. Our findings suggest that besides its important role in ECM production, dermal fibroblasts are also an important source of mitogens and thus are actively involved in skin homeostasis by regulating epidermal keratinocyte proliferation and turnover.

The regulation of epidermal keratinocyte function by paracrine effectors from the dermal microenvironment has been well established in human skin *in vivo*. However, a more complete understanding of these relationships has been delayed due, in part, to a lack of appropriate *in vitro* culture models. In this study we describe a skin equivalent culture model which demonstrates that normal paracrine relationships can be reconstituted *in vitro* and that stromal dermal fibroblasts regulate the growth of human epithelial keratinocytes. Interesting differences in the proliferation of epithelial keratinocytes were noted in the presence or absence of SDF-1. Therefore, skin equivalent culture is a promising model to investigate the interactions between stromal cells and epithelial cells and understand the molecular basis of paracrine effectors from the dermal microenvironment.

To investigate the potential role of the SDF-1/CXCR4 axis, we performed *in vitro* studies using AMD3100, a specific CXCR4-antagonist (Byrne and Sarchio, 2014; Pablos et al., 1999). Our studies confirmed that AMD3100 efficiently blocks SDF-1 interaction with CXCR4 and thus inhibits the activation of ERK and stimulation of keratinocyte proliferation (Fig. 5). AMD3100 has been used clinically as an immunostimulant that mobilizes hematopoietic stem cells in cancer patients (Plerixafor, trade name Mozobil). Our results provide a new therapeutic implication to treat hyperproliferative inflammatory skin diseases using AMD3100. In agreement, AMD3100 has been shown to have beneficial effects in animal models of skin inflammation (Zgraggen et al., 2014) and UV-induced skin cancer (Sarchio et al., 2014). AMD3100 significantly inhibited skin inflammation by reducing inflammatory angiogenesis and inflammatory cell accumulation, and thus preventing skin inflammation and UV-induced skin cancer development. Hence, the SDF-1/CXCR4 chemokine pathway is a novel therapeutic target in the prevention of skin inflammation and UV-induced skin cancer. The role of the SDF-1/CXCR4 axis in skin wound healing is more complex and contradictory (Bollag and Hill, 2013). A single topical application of AMD3100 resulted in accelerated wound healing in diabetic mouse models through enhancement of angiogenesis and vasculogenesis (Nishimura et al., 2012). The studies from Lin et al. further confirmed and extended these findings by identifying that AMD3100 can recruit stem cells into the wound sites and thus accelerates wound healing (Lin et al., 2014). In contrast, other studies have shown that topical application of SDF-1 itself to wound sites enhances healing (Gallagher et al., 2007; Henderson et al., 2011; Rabbany et al., 2010; Sarkar et al., 2011). Nevertheless, the SDF-1/CXCR4 pathway has been implicated in skin wound healing, however, additional investigation of this important SDF-1/CXCR4 axis in skin wound healing is clearly warranted.

**Figure 6:**
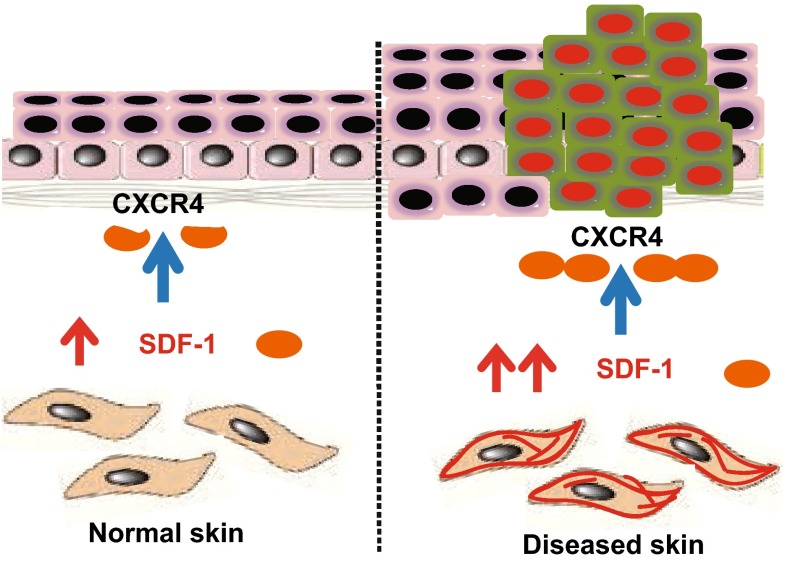
**Proposed model: fibroblast-derived SDF-1 provides a crucial microenvironment for epidermal morphogenesis in both physiological and pathological skin conditions** (see “Discussion” for details)

We propose a working model in which fibroblast-derived SDF-1 provides a crucial microenvironment for epidermal morphogenesis in both physiological and pathological skin conditions (Fig. 6). In physiologically normal human skin, SDF-1 is constitutively and highly expressed in the dermal fibroblasts. Fibroblast-derived SDF-1 promotes keratinocyte proliferation and thus contributes to epidermal turnover and morphogenesis through paracrine signaling. In pathological conditions, SDF-1 expression is further upregulated in dermal fibroblasts and thus contributes to keratinocyte hyperproliferative skin disorders, such as psoriasis and keratinocyte skin cancers. Our data reveal that SDF-1 represents a novel dermal fibroblast-specific factor in the paracrine network, and extremely high levels of SDF-1 provide a crucial microenvironment for epidermal keratinocyte proliferation in both physiological and pathological skin conditions.

## Materials and methods

### Human skin samples

Adult normal human skin samples were obtained by punch biopsy (4 mm) from sun-protected (hip/buttocks) areas of healthy adults (30–40 years), as described previously (Quan et al., 2010). Psoriatic skin samples were obtained by punch biopsy (4 mm) from psoriasis patients. Completely de-identified BCC and SCC samples were obtained from the University of Michigan Cutaneous Oncology Unit. Research involving human subjects was approved by the University of Michigan Institutional Review Board, and informed consent (both written and verbal) was obtained from patients for the use of their skin samples in this research project.

### Laser capture microdissection

For laser capture microdissection (LCM), skin samples embedded in OCT were sectioned (15 µm) and stained with hematoxylin and eosin. Epidermis, dermis, tumor islands, and cancer-associated stromal tissues were captured by LCM (Leica ASLMD system; Leica Microsystems, Wetzlar, Germany), as described previously (Qin et al., 2013). Total cellular RNA was extracted from samples of laser-dissected tissues using an RNeasy® Micro Kit (Qiagen, Chatsworth, CA, USA) according to the manufacturer’s instructions. The quality and quantity of total RNA were determined by Agilent 2100 bioanalyzer (Agilent Technologies, Santa Clara, CA, USA).

### Immunohistology

Immunohistology was performed as described previously (Quan et al., 2010). Briefly, skin samples embedded in OCT were sectioned (7 µm), fixed in 2% paraformaldehyde, permeabilized with 0.5% Triton X-100 in phosphate-buffered saline (PBS), blocked with corresponding serum (5% in PBS), and incubated for one hour at room temperature with SDF-1 (R&D Research, Minneapolis, MN, USA), HSP47, langerin, CD31, CXCR4, and α-smooth muscle actin primary antibodies (Santa Cruz Biotechnology, Santa Cruz, CA), followed by incubation with corresponding secondary antibodies for one hour at room temperature. Between steps, the slides were rinsed for 10 min in Tris buffered saline with 0.1% Triton-X-100 (TBST). All sections were lightly counterstained with haematoxylin. The slides were examined using a digital imaging microscope (Zeiss, Germany). Specificity of staining was determined by substituting corresponding isotype-control immunoglobulins for the primary antibodies.

### Cell culture

Human primary keratinocytes were obtained from Cascade Biologics Inc. (Portland, OR). Adult human primary dermal fibroblasts were prepared from punch biopsies of normal adult buttock skin (aged 22–55 years), as described previously (Fisher et al., 1991). Keratinocytes were maintained in Epilife medium supplemented with keratinocyte growth supplement. Dermal fibroblasts were maintained in Dulbecco’s modified Eagle’s medium (DMEM) supplemented with 10% fetal bovine serum (Invitrogen, Carlsbad, CA). Epilife and DMEM media were supplemented with penicillin (100 U/mL) and streptomycin (100 μg/mL). Cells were cultured in a humidified incubator with 5% CO_2_ at 37°C. Cells were plated at 70%–80% confluence and used one day after plating. Cells were cultured at sub-confluence and utilized between passages 5 and 10. Six days after transfection, cultures were photographed (phase-contract microscopy) and cell numbers determined by hemocytometer.

### RNA isolation and quantitative real-time RT-PCR

Total RNA was extracted using TRizol reagent (Invitrogen, Carlsbad, CA), or RNeasy micro kit (Qiagen, Gaithersburg, MD, USA). cDNA for PCR templates was prepared by reverse transcription of total RNA (100 ng) using Taqman Reverse Transcription kit (Applied Biosystems, Carlsbad, CA, USA). Real-time PCR was performed on a 7700 Sequence Detector (Applied Biosystems, Carlsbad, CA, USA) using Taqman Universal PCR Master Mix Reagents (Applied Biosystems, Carlsbad, CA, USA). SDF-1 real-time PCR primers were designed and purchased from Sigma: sense primer 5′-AGC-CAA-CGT-CAA-GCA-TCT-CAA-3′; antisense primer 5′-AAT-CCA-CTT-TAG-CTT-CGG-GTC-AA-3′. 36B4 primer sequences have been described previously (Quan et al., 2001). The target gene expression levels were determined by relative quantification using the comparative C_T_ method, also known as 2^-∆∆CT^ method (comparative 2–[delta][delta]Ct method). Target gene mRNA expression levels were normalized to the housekeeping gene 36B4 (internal control for quantification), and expressed as a fold change of 36B4.

### Transfection and Western blot analysis

SDF-1siRNAs (#1, 5′-ACGCCAAGGTCGTGGTCGT-3′ position at 94-112, #2, 5′-TGCCGATTCTTCGAAAGCC-3′ position at 183–201, #3, 5′-CTCCAAACTGTGCCCTTCA-3′ position at 244–262) were designed and purchased from Sigma (St. Louis, MO, USA). SDF-1 siRNAs were transiently transfected into human skin dermal fibroblasts by electroporation (Amaxa Nucleofector™, Koeln, Germany). Proteins were prepared from keratinocytes and equal amounts of protein (~50 μg/lane) were analyzed by resolving on 12% sodium dodecyl sulfate-polyacrylamide (SDS) gel electrophoresis. The SDS gels were transferred to polyvinylidenedifluoride membrane, and the membranes were blocked with PBST (0.1% Tween 20 in PBS) containing 5% nonfat milk for one hour at room temperature. Primary antibodies SDF-1 (R&D Research, Minneapolis, MN, USA); α-SMA (Santa Cruz Biotechnology, Santa Cruz, CA, USA); β-actin (Sigma, St. Louis, MO, USA) were incubated with the polyvinylidenedifluoride membrane for one hour at room temperature, after which membranes were washed three times with PBST solution and incubated with appropriate secondary antibodies for one hour at room temperature. After washing three times with PBST, the membranes were developed with ECF (Vistra ECF Western blotting system, GE Health Care, Piscataway, NJ, USA) following the manufacturer’s protocol. The membranes were scanned with a STORM MolecularImager (Molecular Dynamics, Sunnyvale, CA), and the fluorescence intensities of each band were quantified by ImageQuant (GE Health Care, Piscataway, NJ, USA) and normalized using β-actin as a marker for equal protein loading.

### Reconstruction of human skin equivalent culture

Primary adult human dermal fibroblasts between passages 3–8 were used for all experiments. Skin equivalent was prepared based on the well-established standard protocol with minor modification (Boelsma et al., 1999). Briefly, dermal equivalent (fibroblasts-populated collagen lattices) was prepared, as previously described (Fisher et al., 2009). Neutralized rat tail type I collagen (2 mg/mL, BD, Biosciences, Palo Alto, CA, USA) was suspended in medium cocktail [DMEM, NaHCO_3_ (44 mmol/L), L-glutamine (4 mmol/L), Folic Acid (9 mmol/L), and neutralized with 1 N NaOH to pH 7.2]. 1 × 10^5^ cells were suspended in 1 mL collagen and medium cocktail solution and plated on filter inserts. These collagen lattices were placed in an incubator at 37°C for 30 min to allow collagen polymerization. The collagen lattices were then incubated with 2 mL media (DMEM, 10% FBS) at 37°C, 5% CO_2_. One day after dermal equivalent culture, 1 × 10^5^ keratinocytes were seeded on the dermal equivalent collagen lattices. This skin equivalent cultures were grown under submerged conditions in which they were lifted to the air-liquid interface. Cultures were maintained for seven days immersed in a medium composed of MEM (Invitrogen, Carlsbad, CA) supplemented with 10% FBS (Sigma, St Louis, MO), Hydrocortisone (0.4 mg/mL) (Sigma, St Louis, MO), and Cholera Toxin (0.1 nmol/L) (Biomol Int., Plymouth, PA) 50 µg per mL ascorbic acid (50 µg/mL) (Sigma, St Louis, MO), insulin (0.5 µg/mL) (Sigma, St Louis, MO). The culture medium was supplemented with epidermal growth factor (EGF) (10 ng/mL) (BD Biosciences, San Jose, CA, USA) at the time of air exposure. The culture media were renewed every two days. Air-lifted cultures were harvested, embedded in OCT, and cryo-sections were stained with H&E for analysis. In some experiments, the dermal fibroblasts were infected with premade adenovirus SDF-1 (Vigene Biosciences, Rockville, MD, USA) before being embedded in collagen lattices.

### [^3^H]Thymidine incorporation assay

[^3^H]Thymidine incorporation was assayed as follows. Skin equivalent cultures were prepared as described above, and 1 μCi/well [^3^H]methylthymidine (Amersham Pharmacia Biotech, Pittsburgh, PA, USA) was added, and incubation was continued for an additional 16 h. The cells were harvested, and washed three times with ice-cold phosphate-buffered saline, incubated in 10% trichloroacetic acid for 1 h, and incubated with a solution consisting of 0.3 mol/L NaOH and 1% SDS for 2 h. The cells were lysed by vortexing and their radioactivity, which reflects the [^3^H]thymidine incorporation and DNA synthesis, was determined by liquid scintillation counting.

### Statistical analysis

Data are expressed as mean ± SEM. Student’s *t*-test was used to evaluate the statistical differences among the groups. All *P* values are two-tailed, and values less than 0.05 were considered statistically significant.

## Electronic supplementary material

Supplementary material 1 (PDF 100 kb)
