# Supplementary material for: Dermal fibroblast expression of stromal cell-derived factor-1 (SDF-1) promotes epidermal keratinocyte proliferation in normal and diseased skin
Source: Protein Cell. 2015 Aug 22;6(12):890–903. doi: 10.1007/s13238-015-0198-5 (PMC4656211; doi:10.1007/s13238-015-0198-5)
Supplement: Supplementary file 1 — Supplementary material 1 (PDF 100 kb) [file 13238_2015_198_MOESM1_ESM.pdf]

Suppl Figs

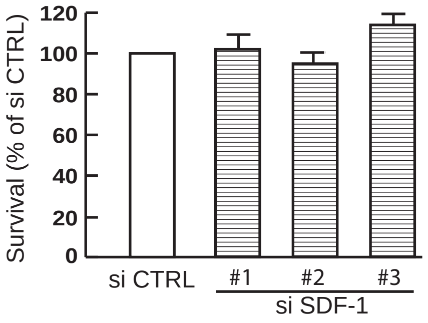

Figure 1. SDF-1 siRNAs off-target effect on cell viability. Dermal fibroblasts were transfected with SDF-1 siRNAs for 2 days, and cell viability was determined by harvesting cells and counting cell numbers with a hemocytometer. Results are means±SEM, N=3.

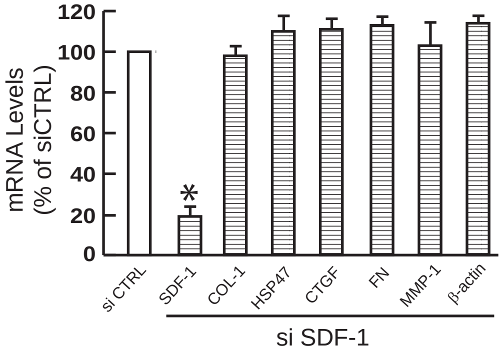

Figure 2. SDF-1 siRNAs off-target effect on mRNA expression. Dermal fibroblasts were transfected with SDF-1 siRNAs for 2 days. mRMA levels of fibroblast-specific genes and housekeeping gene were quantified by real-time RT-PCR, and normalized to the housekeeping gene 36B4, as an internal control for quantification. Results are means±SEM, N=3, *p=0001.

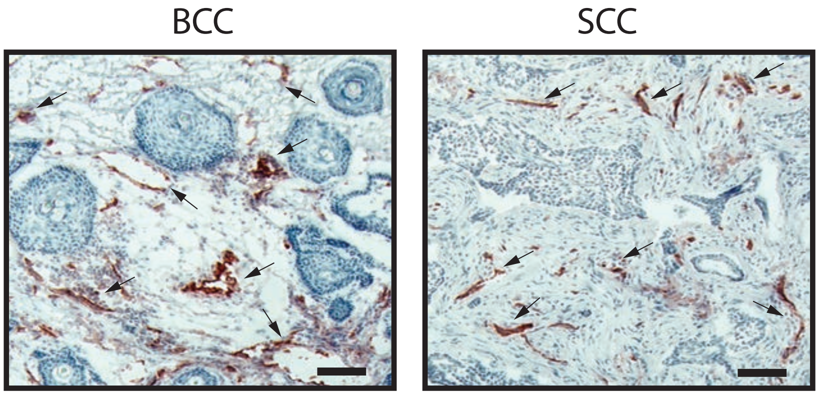

Figure 3. Increased angiogenesis in BCC and SCC. OCT-embedded BCC and SCC skin sections (7μm) were immunostained with CD31, a marker of endothelial cells. Arrows indicate CD31 positive blood vessels. Representative of four individuals. Bar=100μm.
